# Discovery of Targetable Genetic Alterations in NSCLC Patients with Different Metastatic Patterns Using a MassARRAY-Based Circulating Tumor DNA Assay

**DOI:** 10.3390/cells9112337

**Published:** 2020-10-22

**Authors:** Yassine Belloum, Melanie Janning, Malte Mohme, Ronald Simon, Jolanthe Kropidlowski, Alexander Sartori, Darryl Irwin, Manfred Westphal, Katrin Lamszus, Sonja Loges, Sabine Riethdorf, Klaus Pantel, Harriet Wikman

**Affiliations:** 1 Department of Tumor Biology, University Medical Center Hamburg-Eppendorf, 20246 Hamburg, Germany; y.belloum@uke.de (Y.B.); m.janning@uke.de (M.J.); j.kropidlowski@uke.de (J.K.); s.loges@uke.de (S.L.); s.riethdorf@uke.de (S.R.); pantel@uke.de (K.P.); 2 Department of Oncology, Hematology and Bone Marrow Transplantation with Section Pneumology, University Medical Center Hamburg-Eppendorf, 20246 Hamburg, Germany; 3 Division of Personalized Medical Oncology, German Cancer Research Center (DKFZ), 69120 Heidelberg, Germany; 4 Department of Personalized Oncology, University Hospital Mannheim, University of Heidelberg, 68167 Mannheim, Germany; 5 Department of Neurosurgery, University Medical Center Hamburg-Eppendorf, 20246 Hamburg, Germany; M.Mohme@uke.de (M.M.); westphal@uke.de (M.W.); lamszus@uke.uni-hamburg.de (K.L.); 6 Institute of Pathology, University Medical Center Hamburg-Eppendorf, 20246 Hamburg, Germany; r.simon@uke.de; 7 Agena Bioscience GmbH, 22761 Hamburg, Germany; Alexander.Sartori@agenabio.com (A.S.); Darryl.Irwin@agenabio.com (D.I.)

**Keywords:** lung cancer, ctDNA, mutations, liquid biopsy, brain metastases

## Abstract

Circulating tumor DNA (ctDNA) has shown great promise as a minimally invasive liquid biopsy for personalized cancer diagnostics especially among metastatic patients. Here, we used a novel sensitive assay to detect clinically relevant mutations in ctDNA in blood plasma from metastatic non-small cell lung cancer (NSCLC) patients, including patients with a limited oligo–brain metastatic disease. We analyzed 66 plasma samples from 56 metastatic NSCLC patients for 74 hotspot mutations in five genes commonly mutated in NSCLC using a novel MassARRAY-based lung cancer panel with a turnaround time of only 3 days. Mutations in plasma DNA could be detected in 28 out of 56 patients (50.0%), with a variant allele frequency (VAF) ranging between 0.1% and 5.0%. Mutations were detected in 50.0% of patients with oligo–brain metastatic disease, although the median VAF was lower (0.4%) compared to multi-brain metastatic patients (0.9%) and patients with extra-cranial metastatic progression (1.2%). We observed an overall concordance of 86.4% (*n* = 38/44) for *EGFR* status between plasma and tissue. The MassARRAY technology can detect clinically relevant mutations in plasma DNA from metastatic NSCLC patients including patients with limited, oligo–brain metastatic disease.

## 1. Introduction

Non-small cell lung cancers (NSCLC), the most common cause of global cancer-related mortality, are diagnosed in around 40% of patients at late stages in which the primary tumor is inoperable (IIIB and IV) [1]. Knowledge about pathogenic driver mutations is crucial for therapeutic decision-making, since treatment with drugs targeting specific driver mutations improves outcome and quality of life for most patients [2]. However, in many patients with recurrent or progressive disease this information is not available because these patients frequently do not undergo re-biopsies, in particular if the brain is involved as the distant site of metastases. This is mainly due to risk complications associated with tissue biopsy especially at late stages of disease. The occurrence of brain metastases in NSCLC is an increasing clinical problem due to augmented extra-cranial disease control by systemic therapies. Around 40% of advanced stage NSCLC patients will be diagnosed with brain metastases, and the dismal prognosis underlines the urgent need to obtain brain-specific information on therapy targets and resistance mechanisms [3]. Currently, genomic information is most frequently obtained from analysis of the primary tumor or metastases at easily accessible sites. However, brain metastases show a divergent mutation profile from the primary tumor or other metastases [4,5,6]. Thus, future developments in personalized therapy of NSCLC patients will depend on new approaches to obtain tumor DNA from brain metastases for genomic analysis.

In recent years, liquid biopsy has gained importance as novel minimally-invasive source of tumor material for molecular diagnostics that can be complementary to invasive tissues biopsies and to date, cell-free DNA (cfDNA) is perhaps one of the most promising surrogate blood based biomarker candidates for tumor tissue [7,8]. cfDNA refers to extracellular DNA molecules found in body fluids and thought to be released from cells through apoptosis, necrosis and potentially through an active secretion [9,10]. The tumor-derived fraction of cfDNA is commonly referred to as “circulating tumor DNA (ctDNA)”. Analytes in blood such as ctDNA are considered to represent the whole tumor burden at various sites, although different metastases located in different organs might have different shedding rates [11,12]. In fact, due to its noninvasive character, ctDNA might circumvent not only the problem of tissue biopsy but also tumor’ spatial heterogeneity. The dilemma of intra-tumor heterogeneity represents a true limit for personalized medicine approaches because of the reliability on a single tumor tissue biopsy to profile the mutational landscape inherent to each tumor. Gerliner et al. suggested that multiregional biopsy analysis might be required in order to predict the therapeutic outcome and draw a more complete picture of the tumor burden [13]. It is proposed that ctDNA provides a dynamic sampling of somatic alterations capable of representing a larger clonal hierarchy and thus track different treatment responses even at metastatic sites [14,15].

The power of ctDNA analyses in detection of acquired resistance mutations after treatment with 1st and 2nd generation *EGFR* tyrosine kinase inhibitors (TKIs) in NSCLC has been demonstrated in several studies [16]. The detection of ctDNA *EGFR* p.T790M is recommended in current guidelines after progression with *EGFR* TKIs in order to guide treatment initiation with the 3rd generation *EGFR* TKI osimertinib [17].

Although cancer patients generally have higher cfDNA levels compared to healthy individuals, the frequency of ctDNA varies extensively depending on tumor type and disease stage, described to range between 0.01% to 90% [18,19,20,21,22]. In NSCLC patients, the ctDNA levels are generally lower compared to other solid tumors [23]. Several different analytical methods with varying sensitivity and mutation coverage exist today. The sensitivity, specificity, and applicability of the numerous different published ctDNA assays have been reviewed extensively before [7,24]. Thus, highly sensitive and specific ctDNA assays are needed to accurately detect clinically relevant mutations in plasma DNA from brain-metastatic NSCLC patients. For implementation into clinical practice outside of academic institutions, these technologies need to be cost efficient and provide reliable results on a limited panel of druggable mutations within a short turnaround time. Single alterations such as the *EGFR* p.T790M mutation in plasma, can be carried out at very high sensitivity (<0.01% VAF) and cost efficacy by using Digital Droplet PCR (ddPCR) based methods [25,26,27]. However, these methods are restricted to the analysis of a few single mutations, while multiplexing commonly requires NGS-based methods that show variable assay sensitivity and specificity [7,12]. Even though NGS based methods proved their relevance in the clinic and universal genomic sequencing is supported by the clinical community, its implementation in the clinical routine has not been achieved in many countries, mainly due to the high cost of such assays, technical expertise and bioinformatics infrastructure, making it less accessible for a plethora of medical laboratories. 

In this retrospective study, we provide a first proof of principle for a validated ctDNA assay that can detect clinically relevant mutations based on a mass-spectrometry approach in advanced NSCLC patients, including patients with lower tumor burden such as oligo–brain metastatic disease. The MassARRAY system detects in a single multiplex assay 74 hot-spot mutations in five relevant and commonly mutated genes in NSCLC patients with high sensitivity. The present encouraging results qualify this NGS-independent technique as a cost effective, fast, sensitive ctDNA analysis and easily accessible for the medical laboratories. 

## 2. Materials and Methods

### 2.1. Patients and Blood Samples

Blood was drawn from 56 patients with histologically confirmed metastatic (stage IV) NSCLC with a median age of 61 years for both genders. Smoking behavior was not recorded for this cohort. All patients of this retrospective cohort were treated at the University Medical Center of Hamburg, Germany (UKE) (Table 1). All subjects formally consented to the study. Only samples with at least 1.5 mL plasma available and no visual sign of hemolysis were used in this study. The study was approved by the ethics review board of the University of Hamburg (Nr.PV-5392, 06/12/2016, Ärztekammer Hamburg).

In three patients, multiple blood draws were analyzed: From one patient (P01), 9 follow-up samples were available and in two other patients (P09 and P24), two blood samples were available (Figure S1 and Table S3). Figure S1 shows a flow chart on what type of samples and analyses were performed in this study (Figure S1). Thirty-seven patients (66.1%) had brain metastases, 20 of which (35.7%) had metastases only in the brain (oligo–brain metastatic disease) and 16 patients had additional extra central nervous system metastases (multi-brain metastases). For one patient with brain metastases, information of other metastases outside the brain were not recorded (Table 1). We defined oligo-metastatic disease as a purely localized metastases in a single organ, i.e., brain [28]. Therefore, oligo–brain metastatic disease refers to NSCLC patients with an isolated central nervous system (CNS) progression while no extra-CNS disease is recorded. 

In this retrospective cohort, *EGFR* mutation status from tissue analyses was available for 44 patients. As more than half of the patients were collected before 2015 (Table S3), the standard testing was only including *EGFR* and *ALK* for adeno carcinomas. Due to the lack of clinical relevance, *EGFR* mutation status was not routinely assessed in squamous cell cancer patients during the recruitment period of our patients. Nine patients had sensitizing activating mutation in *EGFR* detected in tissue samples (Table S3). In 35 samples, the tissue biopsy was negative for *EGFR* mutations. 

### 2.2. Plasma Isolation and cfDNA Extraction

Plasma was extracted from 7.5 mL of blood drawn in EDTA tubes using a double centrifugation protocol (10 min at 300× *g*, followed by 10 min at 1800× *g*). The cfDNA was extracted using the Circulating Nucleic Acid kit (Qiagen) according to the manufacturer’s protocol. The ctDNA quantity was measured by Qubit Fluorometer (Thermo Fisher) and the quality was assessed by TapeStation (Agilent). 2.3. Mutations analysis using the MassARRAY system

The UltraSEEK™ Lung Panel on the MassARRAY^®^ System (Agena Bioscience, San Diego, CA, USA) analyzes 74 different hot-spot mutations in *EGFR*, *KRAS*, *BRAF*, *ERBB2* and *PIK3CA* (Table S1). This panel was recently validated by using commercial standards in a ring trial comparing different ddPCR, MassARRAY and NGS based assays [29].

The assay consists of a single multiplex PCR reaction targeting specific regions of the five genes, followed by a single base extension relative to the specific mutation using chain terminators. Specific terminating nucleotides are then incorporated only when the mutant allele is present allowing for further enrichment of the mutant signal. The captured and enriched products are then identified using matrix-assisted laser desorption/ionization time-of-flight mass spectrometry as previously described [30]. Data analysis was performed using Typer software version 4.0.26.74 (Agena Bioscience). Normalized intensity was calculated for the signal intensity of the mutant allele, which had been normalized against the capture control peaks found in the spectrum. A value of one means the peak intensity of the observed mutant allele is equal to the peak intensity of the average of the 5 capture control peaks found in the spectrum. The capture control peaks are biotin-labeled, nonreactive oligos, which are added to the extension reaction and used as an internal control for the streptavidin-bead capture and elution of the mutant extension product steps. Mutant allele calls were returned by an automated software report specific for the UltraSEEK Lung Panel and signal-to-noise ratio ≥6 and a z-score ≥7 were considered significant. For allele calling, the reporter algorithm takes an instrument specific baseline for each mutation assay into account. Herein, the assay specific noise is assessed by analyzing a cohort of wild-type samples and mutant call significance was controlled by analyzing commercial mutations controls as a titration of mutant allele frequencies down to the limit of detection (LoD) of 0.1%. 

## 3. Results

### 3.1. Overall Detection of Mutations in Cell Free DNA Using UltraSEEK™ Lung Panel in Advanced NSCLC Patients

**Figure 1:**
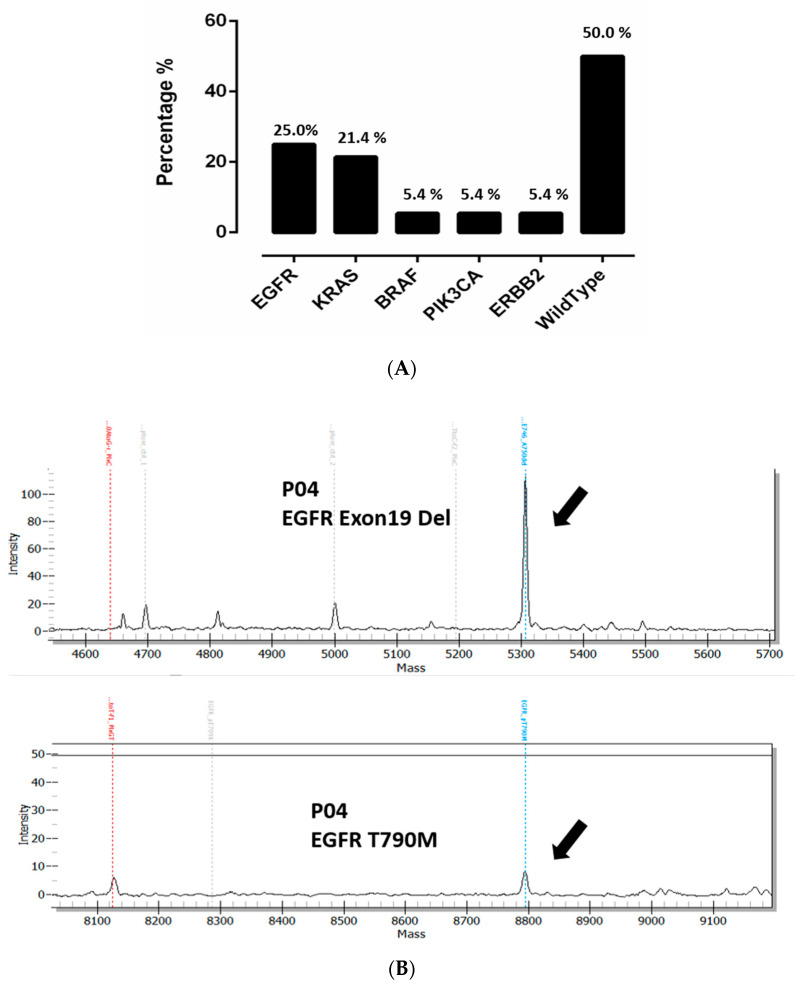
(**A**) Distribution of mutations detected in NSCLC (*n* = 56) patients cfDNA. (**B**) Two MassARRAY plots from patient P04 showing an *EGFR* exon19 deletion and a p.T790M resistance causing mutation.

**Figure 2:**
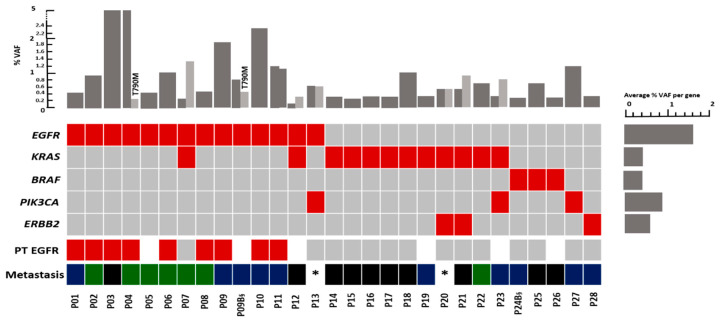
Overview of mutations detected (lower panel) and their variant allele frequency (VAF) (upper panel) in advanced NSCLC patients using the MassARRAY system. When double mutants are detected in one patient, the order of VAF bars are shown in the order of genes in the plot. 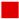 Mutation detected; 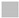 No mutation detected; 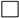 Not determined; 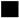 Oligo-brain metastases; 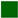 Multi-brain metastases; 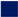 Other metastases; VAF: Variant allele frequency, * Brain metastases in these patients was not documented. §: Follow-up sample, only the first mutation was considered.

Overall, our results showed that mutations could be detected in 28/56 patients (50.0%) using the UltraSEEK™ Lung Panel consisting of 74 different hotspot mutations in five NSCLC associated genes. *EGFR* mutations were detected in 25.0% (14/56), *KRAS* in 21.4% (12/56), *ERBB2* in 5.4% (3/56), *PIK3CA* in 5.4% (3/56), and *BRAF* in 5.4% (3/56) of the patients (Figure 1A and Figure 2). The most common *EGFR* mutations were exon 19 deletion (*EGFR* p.E746_A750Del found in five patients) followed by *EGFR* p.L858R mutations of exon 21 (four patients) (Figure 2 and Table S2). In two *EGFR* positive patients, a resistance causing p.T790M mutation was also detected together with the activating *EGFR* mutation. In patient P04, a p.T790M mutation was detected in addition to exon 19 del (VAF *EGFR* p.E746-A750del 5.0%, VAF *EGFR* p.T790M 0.2%) (Figure 1B). The p.T790M was not observed in the tissue sample taken one month prior to the time of blood draw. However, the patient showed an extra cranial progression from erlotinib one month after the blood draw. From the second patient with a p.T790M mutation (P09), two blood draws were taken. In the first blood draw taken before the beginning of any systemic treatment, an *EGFR* p.E746_A750del was found in both tumor tissue and in the MassARRAY analysis. In a second blood draw 24 months after treatment initiation (patient received both erlotinib and osimertinib), we detected both the *EGFR* p.E746_A750del but also the p.T790M mutation. No tissue biopsy was taken but the p.T790M status was verified in plasma using the Cobas assay (Roche Diagnostics).

The most commonly found *KRAS* mutations were *KRAS* p.G12A/p.G12V (found in five patients) and *KRAS* p.G12C (in four patients). Interestingly in patient P24, the first blood draw at first diagnosis did not show any mutation. Thirteen months later, however, during the second blood draw when the patient had a progressive disease, cfDNA analysis displayed a *BRAF* p.G469A/p.G469V mutation (P24B). Three patients had *PIK3CA* mutations. From these, *PIK3CA* p.H1047R was observed in two patients and *PIK3CA* p.E545K in one patient. *ERBB2* p.A775_G776insYVMA was reported in two patients while one patient displayed *ERBB2* p.G776 > VC mutation (Figure 2 and Table S2).

In eight patients, more than one driver mutation could be found including two patients (P04 and P09B) having *EGFR* activating mutations and the common gatekeeper resistance mutation p.T790M. Figure 2 shows that mutations in *EGFR* and *BRAF* as well as *EGFR* and *ERBB2* were mutually exclusive, which has also been described in primary NSCLC tumors [31]. Two patients (P07 and P12) had detectable *EGFR* and *KRAS* mutations, while *KRAS* and *ERBB2* mutations were identified in two other patients (P20 and P21). One patient (P13) had activating *EGFR* mutation and *PIK3CA* mutation and another patient (P23) displayed a *KRAS* and *PIK3CA* mutation. 

Mutations were detected in plasma NSCLC patients with a VAF ranging from 0.1 to 5.0% (Figure 2 and Table S2). The median VAF in oligo–brain cases was 0.4% (range 0.1–5.0%), while the median VAF in patients with multi-brain metastases was 0.9% (range 0.2–5.0%). The highest median VAF was observed in patients with other metastases 1.2% (range 0.3–2.3%) (Table 2).

### 3.2. Distribution of Mutations in the Plasma of NSCLC Patients with Different Metastatic Patterns

Thirty-seven patients had brain metastases, 16 of which had additional extra-cranial metastases (multi-brain metastases) and 20 patients had the brain as the only site of metastases (oligo–brain metastases). From one patient, the metastatic spread outside the brain was not documented.

In 45.9% (17/37) of the brain metastatic patients (oligo–brain metastases and multi-brain metastases), a mutation in either *EGFR*, *KRAS*, *BRAF* or *ERBB2* ctDNA could be detected. 50.0% of the patients with oligo–brain metastatic disease (*n* = 10) had mutations detected in their blood with a median VAF of 0.4% (Table 2 and Figure 2). *KRAS* was the prevailing mutation present in seven out of the 20 patients with oligo–brain metastases (35.0%). *EGFR* and *BRAF* mutations were detected each in two patients with oligo–brain metastases (10.0%). Among the 16 patients with metastases sites other than in the brain, eight patients (50.0%) had mutations in their blood sample with a median VAF of 1.2%. Four of these patients had detectable *EGFR* mutations (25.0%). Two had *KRAS* mutations (12.5%) and two patients displayed a *PIK3CA* mutation (12.5%) and one *ERBB2* (6.3%). From the 16 multi-brain metastases patients, seven patients (43.8%) had detectable mutations in only *EGFR* and *KRAS* (Table 2). The highest median VAF was observed in the latter setting of brain metastases patients with 1.2%. The total cfDNA amount did not differ between the different groups.

### 3.3. Comparison of EGFR Mutation Status in Tumor Tissue and Plasma

Information about *EGFR* mutation status of the primary tumors was available for 78.6% (*n* = 44/56) of the patients. No other mutations included in the ctDNA analysis were assessed in tissue biopsy from this retrospective patient cohort. By comparing *EGFR* mutations status from plasma (MassARRAY) with matched tumor tissues, an overall concordance of *EGFR* mutational status of 86.4% (38/44) was observed. Six disconcordant cases were identified (P02, P04, P07, P08, P11, and P13 (Table S3)). In most of these cases, the differences in *EGFR* mutational status might have been influenced by the time and treatments between tissue biopsy and liquid biopsy analyses. As it was the case for patient P02, for instance. Here, the tissue biopsy analyses revealed an *EGFR* p.E746-A750del and a resistance causing p.T790M mutation. 5 weeks into treatment with osimertinib (an *EGFR*-TKI specific for p.T790M mutations), a liquid biopsy sample was collected which detected only the *EGFR* p.E746-A750del. The absence of the p.T790M clone in the liquid biopsy samples after 5 weeks into treatment may potentially indicate a response to p.T790M specific treatment with osimertinib. As explained before, a resistance causing p.T790M mutation was detected in the blood of patient P04 taken at extra cranial progression while it was not detected in tissue analysis a month before. In patient P07, the primary biopsy indicated a wild type for *EGFR*, while in the blood sample 5 months later, an *EGFR* mutation (p.L858R) with a low VAF of 0.2% was detected indicating possibly a subclonal origin of the *EGFR* mutation. In patient P13, tissue biopsy indicated a wild type *EGFR*. Two years later, the patient had a progressive disease and blood sample analysis detected a p.L858R *EGFR* and a *PIK3CA* p.H1047R mutation, both with VAF of 0.6%. The tumor tissue was not tested for *PIK3CA.* Unfortunately, confirmatory tissue biopsies for proof were missing for these patients. The concordances between variants found in tissue vs. liquid biopsy along with other clinical information are represented in Table S3. 

## 3.4. Monitoring Patient’s Disease by Tracking Mutations in Plasma ctDNA: A Case Report

**Figure 3:**
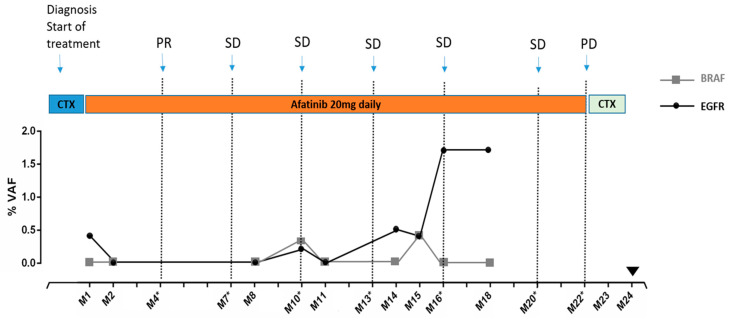
*EGFR* and *BRAF* mutations detection in ctDNA from one patient with known *EGFR* mutation followed at different time points. M: month; *: CT scan. PD: progressive disease. PR: partial remission. SD: stable disease. 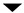: Death of the patient. CTX: chemotherapy. Afatinib: 2nd generation *EGFR* inhibitor.

We analyzed serial blood samples from an 80-year-old patient with a stage IV, multi-metastatic adenocarcinoma of the lung for mutations in ctDNA over a period of 22 months (Figure 3). At first diagnosis, the patient showed metastases at several thoraco–abdominal sites. Due to the high tumor burden, a systemic chemotherapy was started before results of the mutational analyses of tumor tissues were available. These analyses later revealed an *EGFR* p.E746_A750del mutation in the pleura even though in the primary lung tumor tissue analysis, no *EGFR* mutation was detected. Treatment was subsequently switched to afatinib, a 2nd generation *EGFR*-TKI. The first blood draw, performed two weeks after beginning of afatinib treatment, also revealed the *EGFR* p.E746_A750del mutation with 0.4% VAF, concordant to the metastatic pleura cells. Two consecutive blood draws were carried out at months two and eight (M2 and M8), in which no *EGFR* mutation was detected. The decrease and absence of *EGFR* mutations in the blood coincided with a partial response that was detected in the first CT-scan 4 months after initiation of afatinib treatment (M4). The patient continued to have a stable disease based on CT-scans and clinical evaluation for a total of 21 months. During this time however, at month 10, the *EGFR* p.E746_A750del mutation reappeared in the blood sample with a lower VAF: 0.2%. An additional mutation was also detected at month 10: *BRAF* p.V600E with 0.3% VAF (which was not assessed in initial tissue analyses). However, 43 days later at M11, no mutation was detected in the blood. Figure 3 shows that while still having a stable disease based on CT-scans, the *EGFR* p.E746_A750del was detectable with a VAF of 0.5% at month 14 and then VAF decreased slightly to 0.4% at month 15, before increasing again to VAF of 1.7% at month 16. At this time, CT-scans still showed a stable disease and the patients did not experience any new symptoms. CT scans only showed a progressive disease for the first time 6 months after that substantial increase of *EGFR* VAF at month 16. However, at this time point (M22), the physical condition of the patient was deteriorating fast and the patient died shortly afterwards.

## 4. Discussion

Exploring the mutational landscape of brain metastases in individual NSCLC patients is of primary importance for their clinical management. In these patients, biopsies of the brain metastases are seldom taken although a well-known dynamic mutational landscape in brain metastases has been described [5,6]. Here, we analyzed 74 different hotspot mutations in *EGFR*, *KRAS*, *BRAF*, *ERBB2,* and *PIK3CA* genes using a combination of a single multiplexed PCR reaction approach and mass-spectrophotometer based detection platform allowing a fast and cost-effective screening for a relevant number of mutations at high sensitivity. In fact, the sensitivity and the specificity of the assay have recently been validated in a ring trial using commercially available reference material [29], which showed that although sensitivity and specificity were comparable between the different used technologies, the MassARRAY was the assay with the lowest variability in intra-run variant calls. In our current study using patient material, mutations could be detected in 28 of the 56 (50.0%) analyzed metastatic NSCLC patient samples with the variant allele frequency ranging between 0.1% and 5.0%, including patients with oligo–brain metastatic disease. *EGFR* activating mutations in our study were found in 25.0% (14/56) of patients, whilst 21.4% (12/56) of patients displayed a *KRAS* mutation. These data are in line with other studies in Caucasian populations, where, e.g., plasma cfDNA from 23.4% newly diagnosed metastatic NSCLC patients were mutated for *EGFR* and 22.6% had detectable *KRAS* [32,33]. 

Several reports have indicated that the blood–brain barrier (BBB) may inhibit the release of tumor cells or tumor cell products into the bloodstream [34,35,36]. Despite the sensitivity of the MassARRAY technology, we failed to detect ctDNA in approx. 50.0% of oligo–brain metastatic patients, and the VAF was lower compared to patients with multi-brain metastases. This finding is consistent with previous studies using NGS-based assays analyzing 37 genes [37], where 52.0% of oligo–brain metastatic patients had detectable mutations in ctDNA, and the median VAF was lower in patients with oligo–brain metastatic disease compared to patients with multi-brain metastases [37]. Similar obstacles have been also described for primary brain tumors, where somatic alterations in the plasma were also detected in only 50.0% of patients [36]. Besides blockage of ctDNA into the blood by the BBB, oligo–brain metastatic patients have a lower tumor burden than patients with multiple metastases, which might further lower their ctDNA concentrations in blood plasma.

Several studies have shown the superiority of cerebrospinal fluid (CSF) analyses compared to peripheral blood in primary brain tumors, supporting the barrier role of BBB [38,39,40]. A recent paper on NSCLC brain metastatic patients reported that *EGFR* mutations in CSF ctDNA were detected in 57.1% (12/21) of patients, while in only 23.8% (5/21) of paired plasma samples the same mutation could be found [41]. However, the detection rate for blood ctDNA was clearly below the rate found in our present study. In a second similar study, *EGFR* mutations in CSF ctDNA were detected in 63.6% (14/22) against 45.5% (10/22) of paired plasma samples [42]. Future studies using both CSF and plasma might be warranted. In general, obtaining CSF is more invasive than drawing blood, which might hamper the clinical use of CSF for sequential monitoring of tumor responses to therapy.

In three patients, follow-up samples were available and exemplified the power of longitudinal testing. In one patient, nine samples could be collected over a period of 22 months. Here the *EGFR* mutation was detected at initial diagnosis but then remained undetectable during a long stable disease phase. However, at month 16 when the CT-scan still did not show any progression, the *EGFR* mutation was again clearly detected in ctDNA. The CT-scan detected relapse 6 months later, two months before the patient’s death. Our data thus support the use of ctDNA and sequential sampling to track upcoming resistance/relapse, and consequently upholds previous studies investigating the clinical relevance of blood based p.T790M mutation detection for NSCLC patients under *EGFR* TKI treatment [16,43]. Similarly, we recently showed, for the first time, using cfDNA plasma that a *MET* amplification can cause a resistance in *ALK* positive NSCLC patients receiving crizonitib [44]. Prospective clinical studies still need to show, whether tracking mutations on plasma DNA is as relevant as tumor biopsy analysis for making treatment decisions.

Some discrepancies between plasma and tissue DNA analyses are commonly observed in NSCLC and other tumor entities [25,37,45]. Here the MassARRAY based technology showed an overall concordance rate of 86.4% between the *EGFR* mutational status in tumor tissue vs. liquid biopsy. Besides technical issues, “private” plasma DNA mutations might support the overarching hypothesis of liquid biopsy: Blood functions as a pool of tumor cells and tumor cell-products released not only from the primary lesion but also from metastatic sites and therefore provides a more comprehensive information than a single tissue biopsy [11,12]. However, clearly larger studies including matched information of all mutations in tumor and plasma are needed to validate the ultimate sensitivity of this assay. Furthermore, although this study showed the feasibility of detecting point mutations at low VAF, it did not assess the clinically important *ALK*, *ROS*, *RET*, *NTRK,* translocations or *MET* exon 14 mutations. The mass-spectrometric approach is, however, adaptable to detect additional point mutations beyond the panel used in the present study, whereas translocations are in general harder to detect in plasma [46]. Despite these limitations, the benefits of this assay include its cost effectiveness and low turnaround time combined without the need for complex data analysis, bioinformatics pipelines or large data storage capacity. Furthermore, the MassARRAY system is a flexible platform allowing a broad range of different clinical applications such as HPV detection [47], tumor profiling [48], pharmacogenetics (SNP) analyses [49], sample qualification [50], and SARS-CoV-2 testing [51], thus being well suitable and accessible for a various medical laboratories with clearly lower running costs compared to NGS based analyses.

## 5. Conclusions

Real-time monitoring of the changeable mutational landscape of metastatic patients by liquid biopsy approaches can be of great aid for their optimal clinical management. Here we show that the MassARRAY-based assay is a cost effective method that provided information on druggable mutations even in patients with limited, oligo–brain metastatic disease. We could furthermore show that by using longitudinal ctDNA monitoring we could track upcoming resistance and relapse before conventional imaging, showing that the MassARRAY-based assay is providing clinical meaningful results in an efficient and sensitive manner.

## Figures and Tables

**Table 1:** Clinical characteristics of non-small cell lung cancer (NSCLC) patients’ cohort.

Characteristics		Number	Percentage
Gender	Male	26	46.4%
	Female	30	53.6%
Histology	Adeno ca.	49	87.5%
	Squamous cell ca.	5	8.9%
	other	2	3.6%
*EGFR* in tissue *	Mutant	9	20.5%
	Wild type	35	79.5%
Disease stage	First diagnosis	39	69.6%
	Progressive disease	16	28.6%
	Complete response	1	1.8%
Metastases **	Brain metastases	37	66.1%
	Other metastases than brain	16	28.6%
	Unknown	3	5.5%
Brain- Metastases ***	Oligo-brain metastases	20	54.0%
	Multi-brain metastases	16	43.2%
	Unknown	1	2.7%

* *EGFR* tissue status was not assessed in 12 patients. ** For three metastatic patients, the metastatic spread within the brain was not documented at the time of blood draw. *** From one brain metastatic patient, the metastatic spread outside the brain was not documented.

**Table 2:** Prevalence of detected mutations in circulating tumor DNA (ctDNA) samples.

	BRAF	EGFR	KRAS	ERBB2	PIK3CA	Number of Pts	Median VAF
	*n* (%)	*n* (%)	*n* (%)	*n* (%)	*n* (%)	with Mutation (%)	of All Mutations
Oligo-brain metastases (*n* = 20) *	2 (10.0%)	2 (10.0%)	7 (35.0%)	1 (5.0%)	0	10 (50.0%)	0.4
Multi-brain metastases (*n* = 16) **	0	6 (37.5%)	2 (12.5%)	0	0	7 (43.8%)	0.9
Other metastases (*n* = 16) ***	0	4 (25.0%)	2 (12.5%)	1 (6.3%)	2 (12.5%)	8 (50.0%)	1.2

* Patient P12 had both an EGFR and a KRAS mutation, patient P21 had a KRAS and an ERBB2 mutation. ** Patient P07 had EGFR and KRAS mutations. *** Patient P23 had KRAS and PIK3CA mutations.

## Supplementary Materials

The following are available online at https://www.mdpi.com/2073-4409/9/11/2337/s1, Figure S1: Flowchart of the study cohort. Table S1: Targets genes in the UltraSEEK lung panel. Table S2: Detected mutations and the VAF in plasma cfDNA from NSCLC patients. Table S3: *EGFR* mutation status in matched plasma and tumor tissue of advanced NSCLC patients.

## References

[1] Bray F., Me J.F., Soerjomataram I., Siegel R.L., Torre L.A., Jemal A. (2018). Global cancer statistics 2018: GLOBOCAN estimates of incidence and mortality worldwide for 36 cancers in 185 countries. CA Cancer J. Clin..

[2] Heigener D.F., Kerr K.M., Laing G.M., Mok T., Moiseyenko F.V., Reck M. (2019). Redefining Treatment Paradigms in First-line Advanced Non-Small-Cell Lung Cancer. Clin. Cancer Res..

[3] Boire A., Brastianos P.K., Garzia L., Valiente M. (2020). Brain metastasis. Nat. Rev. Cancer.

[4] Hohensee I., Lamszus K., Riethdorf S., Meyer-Staeckling S., Glatzel M., Matschke J., Witzel I., Westphal M., Brandt B., Müller V. (2013). Frequent Genetic Alterations in EGFR- and HER2-Driven Pathways in Breast Cancer Brain Metastases. Am. J. Pathol..

[5] Brastianos P.K., Carter S.L., Santagata S., Cahill D.P., Taylor-Weiner A., Jones R.T., van Allen E.M., Lawrence M.S., Horowitz P.M., Cibulskis K. (2015). Genomic Characterization of Brain Metastases Reveals Branched Evolution and Potential Therapeutic Targets. Cancer Discov..

[6] Shih D.J.H., Nayyar N., Bihun I., Dagogo-Jack I., Gill C.M., Aquilanti E., Bertalan M., Kaplan A., D’Andrea M.R., Chukwueke U. (2020). Genomic characterization of human brain metastases identifies drivers of metastatic lung adenocarcinoma. Nat. Genet..

[7] Heitzer E., Haque I.S., Roberts C.E.S., Speicher M.R. (2018). Current and future perspectives of liquid biopsies in genomics-driven oncology. Nat. Rev. Genet..

[8] Marques J.F., Reis J.P., Fernandes G., Hespanhol V., Machado J.C., Costa J.L. (2019). Circulating Tumor DNA: A Step into the Future of Cancer Management. Acta Cytol..

[9] Wan J.C.M., Massie C., Garcia-Corbacho J., Mouliere F., Brenton J.D., Caldas C.M., Pacey S., Baird R., Rosenfeld N. (2017). Liquid biopsies come of age: Towards implementation of circulating tumour DNA. Nat. Rev. Cancer.

[10] Keller L., Belloum Y., Wikman H., Pantel K. (2020). Clinical relevance of blood-based ctDNA analysis: Mutation detection and beyond. Br. J. Cancer.

[11] Keller L., Pantel K. (2019). Unravelling tumour heterogeneity by single-cell profiling of circulating tumour cells. Nat. Rev. Cancer.

[12] Pantel K., Alix-Panabières C. (2019). Liquid biopsy and minimal residual disease—latest advances and implications for cure. Nat. Rev. Clin. Oncol..

[13] Gerlinger M., Rowan A.J., Horswell S., Larkin J., Endesfelder D., Gronroos E., Martinez P., Matthews N., Stewart A., Tarpey P. (2012). Intratumor Heterogeneity and Branched Evolution Revealed by Multiregion Sequencing. N. Engl. J. Med..

[14] Murtaza M., Dawson S.-J., Pogrebniak K., Rueda O.M., Provenzano E., Grant J., Chin S.-F., Tsui D.W.Y., Marass F., Gale D. (2015). Multifocal clonal evolution characterized using circulating tumour DNA in a case of metastatic breast cancer. Nat. Commun..

[15] Marusyk A., Janiszewska M., Polyak K. (2020). Intratumor Heterogeneity: The Rosetta Stone of Therapy Resistance. Cancer Cell.

[16] Oxnard G.R., Thress K.S., Alden R.S., Lawrance R., Paweletz C.P., Cantarini M., Yang J.C.-H., Barrett J.C., Jänne P.A. (2016). Association Between Plasma Genotyping and Outcomes of Treatment with Osimertinib (AZD9291) in Advanced Non–Small-Cell Lung Cancer. J. Clin. Oncol..

[17] Planchard D., Popat S., Kerr K., Novello S., Smit E., Faivre-Finn C., Mok T., Reck M., van Schil P., Hellmann M. (2018). Metastatic non-small cell lung cancer: ESMO Clinical Practice Guidelines for diagnosis, treatment and follow-up. Ann. Oncol..

[18] Schwarzenbach H., Alix-Panabières C., Müller I., Letang N., Vendrell J.P., Rebillard X., Pantel K. (2009). Cell-free Tumor DNA in Blood Plasma as a Marker for Circulating Tumor Cells in Prostate Cancer. Clin. Cancer Res..

[19] Schwarzenbach H., Hoon D.S.B., Pantel K. (2011). Cell-free nucleic acids as biomarkers in cancer patients. Nat. Rev. Cancer.

[20] Schwarzenbach H., Stoehlmacher J., Pantel K., Goekkurt E. (2008). Detection and Monitoring of Cell-Free DNA in Blood of Patients with Colorectal Cancer. Ann. N. Y. Acad. Sci..

[21] Salvianti F., Pinzani P., Verderio P., Ciniselli C.M., Massi D., de Giorgi V., Grazzini M., Pazzagli M., Orlando C. (2012). Multiparametric Analysis of Cell-Free DNA in Melanoma Patients. PLoS ONE.

[22] Qin Z., Ljubimov V.A., Zhou C., Tong Y., Liang J. (2016). Cell-free circulating tumor DNA in cancer. Chin. J. Cancer.

[23] Smith C.G., Moser T., Mouliere F., Field-Rayner J., Eldridge M., Riediger A.L., Chandrananda D., Heider K., Wan J.C.M., Warren A.Y. (2020). Comprehensive characterization of cell-free tumor DNA in plasma and urine of patients with renal tumors. Genome Med..

[24] Elazezy M., Joosse S.A. (2018). Techniques of using circulating tumor DNA as a liquid biopsy component in cancer management. Comput. Struct. Biotechnol. J..

[25] Steendam C.M., Atmodimedjo P., de Jonge E., Paats M.S., van der Leest C., Hoop E.O.D., Jansen M.P., del Re M., von der Thüsen J.H., Dinjens W.N. (2019). Plasma Cell-Free DNA Testing of Patients with EGFR Mutant Non–Small-Cell Lung Cancer: Droplet Digital PCR Versus Next-Generation Sequencing Compared with Tissue-Based Results. JCO Precis. Oncol..

[26] Jiang X., Liu W., Zhu X., Xu X. (2019). Evaluation of EGFR mutations in NSCLC with highly sensitive droplet digital PCR assays. Mol. Med. Rep..

[27] Zhu G., Ye X., Dong Z., Lu Y.C., Sun Y., Liu Y., McCormack R., Gu Y., Liu X. (2015). Highly Sensitive Droplet Digital PCR Method for Detection of EGFR-Activating Mutations in Plasma Cell–Free DNA from Patients with Advanced Non–Small Cell Lung Cancer. J. Mol. Diagn..

[28] Hellman S., Weichselbaum R.R. (1995). Oligometastases. J. Clin. Oncol..

[29] Weber S., Spiegl B., Perakis S., Ulz C.M., Abuja P.M., Kashofer K., van der Leest P., Azpurua M.A., Tamminga M., Brudzewsky D. (2020). Technical Evaluation of Commercial Mutation Analysis Platforms and Reference Materials for Liquid Biopsy Profiling. Cancers.

[30] Mosko M.J., Nakorchevsky A.A., Flores E., Metzler H., Ehrich M., Boom D.J.V.D., Sherwood J.L., Nygren A.O. (2016). Ultrasensitive Detection of Multiplexed Somatic Mutations Using MALDI-TOF Mass Spectrometry. J. Mol. Diagn..

[31] Collison A.E., Campbell J.D., Brooks A.N., Berger A.H., Lee W., Chmielecki J., Beer D.G., Cope L., Creighton C.J., Danilova L. (2014). Comprehensive molecular profiling of lung adenocarcinoma. Nat. Cell Biol..

[32] Sacher A.G., Paweletz C., Dahlberg S.E., Alden R.S., O’Connell A., Feeney N., Mach S.L., Jänne P.A., Oxnard G.R. (2016). Prospective Validation of Rapid Plasma Genotyping for the Detection ofEGFRandKRASMutations in Advanced Lung Cancer. JAMA Oncol..

[33] Oxnard G.R., Paweletz C.P., Kuang Y., Mach S.L., O’Connell A., Messineo M.M., Luke J.J., Butaney M., Kirschmeier P., Jackman D.M. (2014). Noninvasive Detection of Response and Resistance in EGFR-Mutant Lung Cancer using Quantitative Next-Generation Genotyping of Cell-Free Plasma DNA. Clin. Cancer Res..

[34] Hanssen A., Riebensahm C., Mohme M., Joosse S.A., Velthaus J.-L., Berger L.A., Bernreuther C., Glatzel M., Loges S., Lamszus K. (2018). Frequency of Circulating Tumor Cells (CTC) in Patients with Brain Metastases: Implications as a Risk Assessment Marker in Oligo-Metastatic Disease. Cancers.

[35] Riebensahm C., Joosse S.A., Mohme M., Hanssen A., Matschke J., Goy Y., Witzel I., Lamszus K., Kropidlowski J., Petersen C. (2019). Clonality of circulating tumor cells in breast cancer brain metastasis patients. Breast Cancer Res..

[36] Piccioni D.E., Achrol A.S., Kiedrowski L.A., Banks K.C., Boucher N., Barkhoudarian G., Kelly D.F., Juarez T., Lanman R.B., Raymond V.M. (2019). Analysis of cell-free circulating tumor DNA in 419 patients with glioblastoma and other primary brain tumors. CNS Oncol..

[37] Aldea M., Hendriks L., Mezquita L., Jovelet C., Planchard D., Auclin E., Remon J., Howarth K., Benitez J.C., Gazzah A. (2020). Circulating Tumor DNA Analysis for Patients with Oncogene-Addicted NSCLC with Isolated Central Nervous System Progression. J. Thorac. Oncol..

[38] Miller A.M., Shah R.H., Pentsova E.I., Pourmaleki M., Briggs S., Distefano N., Zheng Y., Skakodub A., Mehta S.A., Campos C. (2019). Tracking tumour evolution in glioma through liquid biopsies of cerebrospinal fluid. Nat. Cell Biol..

[39] Pan C., Diplas B.H., Chen X., Wu Y., Xiao X., Jiang L., Geng Y., Xu C., Sun Y., Zhang P. (2018). Molecular profiling of tumors of the brainstem by sequencing of CSF-derived circulating tumor DNA. Acta Neuropathol..

[40] Zhao Z., Zhang C., Li M., Shen Y., Feng S., Liu J., Li F., Hou L., Chen Z., Jiang J. (2020). Applications of cerebrospinal fluid circulating tumor DNA in the diagnosis of gliomas. Jpn. J. Clin. Oncol..

[41] Ma C., Yang X., Xing W., Yu H., Si T., Guo Z. (2020). Detection of circulating tumor DNA from non-small cell lung cancer brain metastasis in cerebrospinal fluid samples. Thorac. Cancer.

[42] Huang R., Xu X., Li D., Chen K., Zhan Q., Ge M., Zhou X., Liang X., Guan M. (2019). Digital PCR-Based Detection of EGFR Mutations in Paired Plasma and CSF Samples of Lung Adenocarcinoma Patients with Central Nervous System Metastases. Target. Oncol..

[43] Kuang Y., Rogers A., Yeap B.Y., Wang L., Makrigiorgos M., Vetrand K., Thiede S., Distel R.J., Janne P.A. (2009). Noninvasive Detection of *EGFR* T790M in Gefitinib or Erlotinib Resistant Non–Small Cell Lung Cancer. Clin. Cancer Res..

[44] Berger L.A., Janning M., Velthaus J.L., Ben-Batalla I., Schatz S., Falk M., Iglauer P., Simon R., Cao R., Forcato C. (2018). Identification of a High-Level MET Amplification in CTCs and cfTNA of an ALK-Positive NSCLC Patient Developing Evasive Resistance to Crizotinib. J. Thorac. Oncol..

[45] Karachaliou N., Casas C.M.D.L., Queralt C., de Aguirre I., Melloni B., Cardenal F., Garcia-Gomez R., Massuti B., Sánchez J.M., Porta R. (2015). Association ofEGFRL858R Mutation in Circulating Free DNA with Survival in the EURTAC Trial. JAMA Oncol..

[46] Vendrell J.A., Mau-Them F.T., Béganton B., Godreuil S., Coopman P., Solassol J. (2017). Circulating Cell Free Tumor DNA Detection as a Routine Tool forLung Cancer Patient Management. Int. J. Mol. Sci..

[47] Kriegsmann M., Wandernoth P., Lisenko K., Casadonte R., Longuespée R., Arens N., Kriegsmann J. (2016). Detection of HPV subtypes by mass spectrometry in FFPE tissue specimens: A reliable tool for routine diagnostics. J. Clin. Pathol..

[48] Toomey S., Carr A., Mezynski M.J., Elamin Y., Rafee S., Cremona M., Morgan C., Madden S., Abdul-Jalil K.I., Gately K. (2020). Identification and clinical impact of potentially actionable somatic oncogenic mutations in solid tumor samples. J. Transl. Med..

[49] Scott S.A., Scott E.R., Seki Y., Chen A.J., Wallsten R., Obeng A.O., Botton M.R., Cody N., Shi H., Zhao G. (2020). Development and Analytical Validation of a 29 Gene Clinical Pharmacogenetic Genotyping Panel: Multi-Ethnic Allele and Copy Number Variant Detection. Clin. Transl. Sci..

[50] Miller J.K., Buchner N., Timms L., Tam S., Luo X., Brown A.M., Pasternack D., Bristow R.G., Fraser M., Boutros P.C. (2014). Use of Sequenom sample ID Plus(R) SNP genotyping in identification of FFPE tumor samples. PLoS ONE.

[51] Wandernoth P., Kriegsmann K., Groh-Mohanu C., Daeumer M., Gohl P., Harzer O., Kriegsmann M., Kriegsmann J. (2020). Detection of Severe Acute Respiratory Syndrome Coronavirus 2 (SARS-CoV-2) by Mass Spectrometry. Viruses.

