# Supplementary material for: Discovery of Targetable Genetic Alterations in NSCLC Patients with Different Metastatic Patterns Using a MassARRAY-Based Circulating Tumor DNA Assay
Source: Cells. 2020 Oct 22;9(11):2337. doi: 10.3390/cells9112337 (PMC7690267; doi:10.3390/cells9112337)
Supplement: Supplementary file 1 [file cells-09-02337-s001.zip › cells-953206 suppl-re/Figure S1.pptx]

## Slide 1
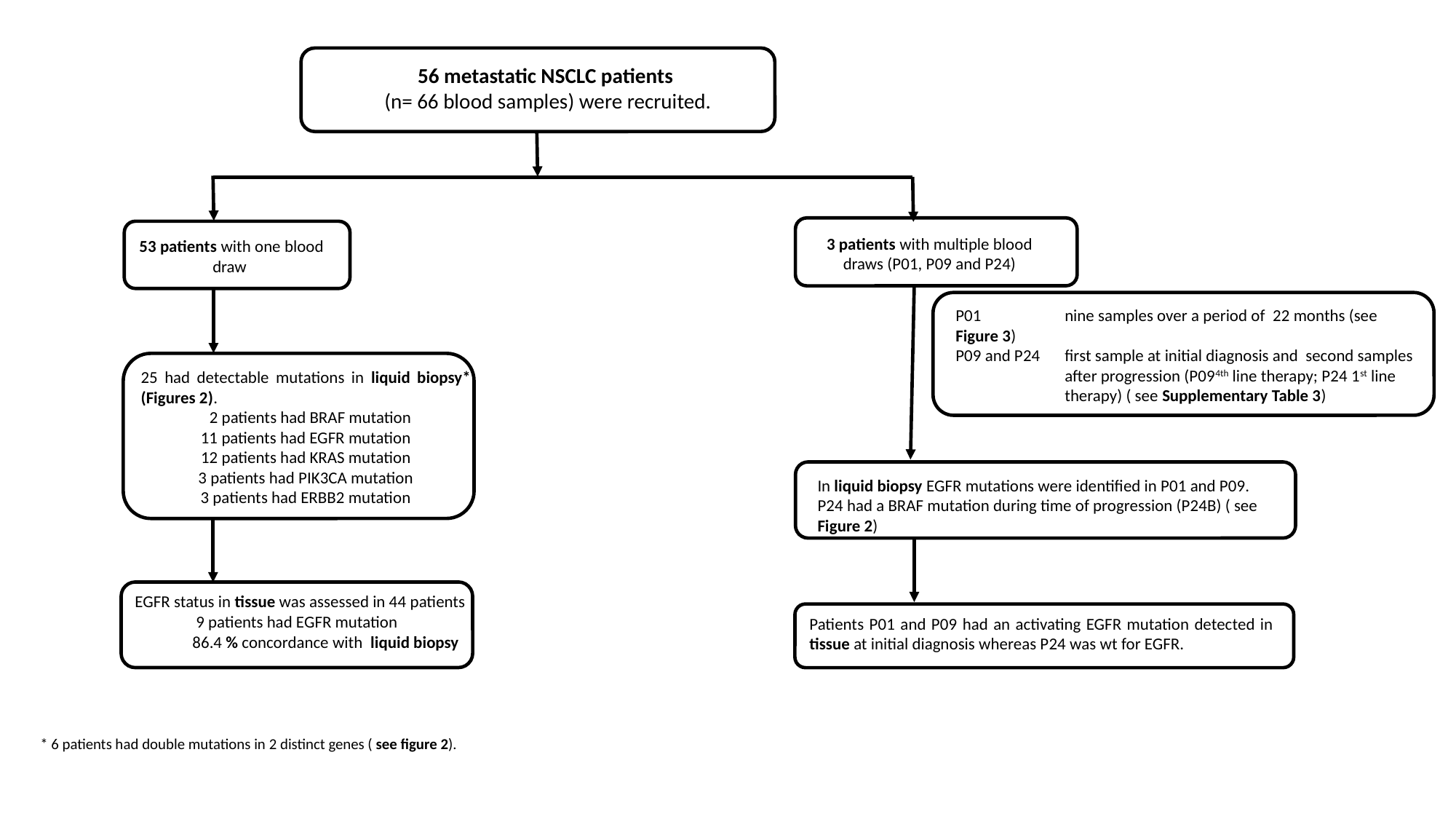

56 metastatic NSCLC patients
(n= 66 blood samples) were recruited.
3 patients with multiple blood draws (P01, P09 and P24)
53 patients with one blood draw
P01	nine samples over a period of 22 months (see 	Figure 3)
P09 and P24	first sample at initial diagnosis and second samples 	after progression (P094th line therapy; P24 1st line 	therapy) ( see Supplementary Table 3)
25 had detectable mutations in liquid biopsy* (Figures 2).
 2 patients had BRAF mutation
11 patients had EGFR mutation
12 patients had KRAS mutation
3 patients had PIK3CA mutation
3 patients had ERBB2 mutation
In liquid biopsy EGFR mutations were identified in P01 and P09. P24 had a BRAF mutation during time of progression (P24B) ( see Figure 2)
EGFR status in tissue was assessed in 44 patients
 9 patients had EGFR mutation
 86.4 % concordance with liquid biopsy
Patients P01 and P09 had an activating EGFR mutation detected in tissue at initial diagnosis whereas P24 was wt for EGFR.
* 6 patients had double mutations in 2 distinct genes ( see figure 2).
* P01 with several FUP will be addressed alone.
