# Supplementary material for: Discovery of Targetable Genetic Alterations in NSCLC Patients with Different Metastatic Patterns Using a MassARRAY-Based Circulating Tumor DNA Assay
Source: Cells. 2020 Oct 22;9(11):2337. doi: 10.3390/cells9112337 (PMC7690267; doi:10.3390/cells9112337)
Supplement: Supplementary file 1 [file cells-09-02337-s001.zip › cells-953206 suppl-re/Table S1 and S2_Updated.pdf]

Supplementary Table S1: targets genes in the UltraSEEK lung panel

**Gene**	**UltraSEEK^{TM} Lung Panel Coverage (Missense mutations) Agena Bioscience**	**# of mutations**
**BRAF**	Codon 469 (exon11) and codons 594, 600 (exon15)	4
**EGFR**	E709A, E709G, E709K, E709V, G719A, G719D, G719S, G719C, S768I, T790M, L858R, L861Q; L861R; C797S; Exon19 indels, Exon20 insertions	46
**KRAS**	Codons 12, 13 (exon 2) and 61 (exon 3)	16
**ERBB2**	Exon 20 insertions	4
**PIK3CA**	Codons 542, 545 (exon9), codon 1047 of (exon20)	4
**TOTAL**	**5 genes**	74

Supplementary Table S2: detected mutations and the VAF in plasma cfDNA from NSCLC patients

Pat	Amino Acid change	% VAF
**P01**	EGFR_p.E746_A750Del	0.4
**P02**	EGFR_p.E746_A750Del	0.9
**P03**	EGFR_p.E746_A750Del	5
**P04**	EGFR_p.E746_A750Del	5
	T790M	0.2
**P05**	EGFR_p.L858R	0.4
**P06**	EGFR_p.L858R	1
**P07**	EGFR_p.L858R	0.2
	KRAS_p.G12A/p.G12V	1.3
**P08**	EGFR_p.E709A	0.4
**P09**	EGFR_p.E746_A750Del	1.8
**P09B**	EGFR_p.E746_A750Del	0.8
	T790M	0.4
**P10**	L.747_T751del	2.3
**P11**	EGFR_pL861Q/P.L861R	1.2
	EGFR_p.L747_A750>P	1.1
**P12**	EGFR_p.N771>TH	0.1
	KRAS_p.G12A/p.G12V	0.3
**P13**	EGFR_p.L858R	0.6
	PIK3CA_p.H1047R	0.6
**P14**	KRAS_p.G12C	0.3
**P15**	KRAS_p.G12A/p.G12V	0.2
**P16**	KRAS_p.G12A/p.G12V	0.3
**P17**	KRAS_p.G12A/p.G12V	0.3
**P18**	KRAS_p.G12R	1
**P19**	KRAS_p.G12C	0.3
**P20**	KRAS_p.G12C	0.5
	ERBB2_p.A775_G776insYVMA	0.5
**P21**	KRAS_p.G12F	0.5
	ERBB2_p.A775_G776insYVMA	0.9
**P22**	KRAS_p.G13D	0.7
**P23**	KRAS_p.G12C	0.3
	PIK3CA_p.H1047R	0.8
**P24B**	BRAF_p.G469A/p.G469V	0.2
**P25**	BRAF_p.G469A/p.G469V	0.7
**P26**	BRAF_p.D594G	0.2

**P27**	PIK3CA_p.E545K	1.2
**P28**	ERBB2_p.G776>VC	0.3
